# Genome-Wide Association Analysis Identifies Genomic Regions and Candidate Genes for Growth and Fatness Traits in Diannan Small-Ear (DSE) Pigs

**DOI:** 10.3390/ani13091571

**Published:** 2023-05-08

**Authors:** Mei Liu, Qun Lan, Long Yang, Qiuchun Deng, Taiyun Wei, Heng Zhao, Peiya Peng, Xiaoding Lin, Yuhan Chen, Haiming Ma, Hongjiang Wei, Yulong Yin

**Affiliations:** 1 College of Animal Science and Technology, Hunan Agricultural University, Changsha 410128, China; mei.liu@hunau.edu.cn (M.L.);; 2 Yunnan Province Key Laboratory for Porcine Gene Editing and Xenotransplantation, Yunnan Agricultural University, Kunming 650201, China; 3 Guangdong Laboratory for Lingnan Modern Agriculture, Guangzhou 510642, China; 4 Key Laboratory of Agro-Ecological Processes in Subtropical Region, Institute of Subtropical Agriculture, Chinese Academy of Sciences, Changsha 410125, China

**Keywords:** Diannan small-ear pig, candidate gene, growth trait, fatness trait, GWAS

## Abstract

**Simple Summary:**

Growth and fatness traits are economically important in pig farming. The exploration of underlying genetic architecture for vital phenotypes is helpful in speeding up the process of genetic improvement for different pig breeds. Hence, for Diannan small-ear (DSE) pig, eight phenotypic traits including six body measurement traits and two fatness traits were examined. Based on the Geneseek Porcine 50K SNP Chip data, single nucleotide polymorphisms (SNPs) were detected in a DSE pig population. Through a genome-wide association study, some candidate genes were detected potentially related to the traits of interest. These findings help to understand the genetic basis of porcine growth traits could be used in future pig breeding schemes.

**Abstract:**

In the livestock industry, the growth and fatness traits are directly related to production efficiency and economic profits. As for Diannan small-ear (DSE) pigs, a unique indigenous breed, the genetic architecture of growth and fatness traits is still elusive. The aim of this study was to search the genetic loci and candidate genes associated with phenotypic traits in DSE pigs using GWAS based on the Geneseek Porcine 50K SNP Chip data. A total of 22,146 single nucleotide polymorphisms (SNPs) were detected in 265 DSE pigs and used for Genome-wide association studies (GWAS) analysis. Seven SNPs were found to be associated with back height, chest circumference, cannon bone circumference, and backfat thickness at the suggestive significance level. Based on gene annotation results, these seven SNPs were, respectively, mapped to the following candidate genes, *VIPR2*, *SLC10A2*, *NUCKS1*, *MCT1*, *CHCHD3*, *SMOX*, and *GPR1*, which are mainly involved with adipocyte differentiation, lipid metabolism, skeletal muscle development, and average daily weight gain. Our work offers novel insights into the genetic architecture of economically important traits in swine and may play an important role in breeding using molecular markers in the DSE breed.

## 1. Introduction

Growth and fatness traits are economically important in the global pig breeding industry. Pig growth and fatness traits, such as backfat thickness, body weight, body length, and chest circumference, are the key traits of interest when making a breeding program. This is because they have an important relationship with productivity. For instance, backfat thickness was regarded as the evaluation indicator for carcass lean percentage [1]. A larger physical size (body weight and body length) indicates more total meat content. These phenotypic traits are regulated by both genetic determinants and environmental factors, exhibiting low to moderate heritability [2]. During the process of natural selection, Chinese indigenous pig breeds exhibited many excellent characteristics and large phenotypic variation [3]. For example, Tibetan pigs have a smaller physical size and exhibit heritable adaptation to the plateau area [4]. Erhualian pigs are well known for their universally high fertility [5]. Min pigs, which inhabit the northeast area in China, are well known for their prominent characteristics, such as tolerance to low-quality roughage, superior meat quality, and high intramuscular fat (IMF) [6]. As a specific pig breed of China, the Diannan small-ear (DSE) pig (Figure S1) is primarily raised in the southern region of Yunnan province, which has a subtropical climate, and is characterized by more fat deposition, lower growth rate, better meat quality, and higher adaptability to adverse environmental conditions [7]. Nevertheless, the shared disadvantage of the local breeds including DSE pig is their high fat deposition and variation in individual size, leading to a low feed conversion rate and slow growth. Therefore, it is important to identify candidate genes or genetic variants that are related to pig growth and fatness traits.

During the last decade, breeders specifically focused on improving the individual size and decreasing backfat thickness to elevate production efficiency. Genome-wide association studies (GWAS) represent a powerful and ubiquitous tool for investigating the genetic basis for phenotypic traits of interest, and it was widely used to assess the genetic architecture of growth and fatness traits in various swine populations, including Chinese local breeds such as Erhualian [8] and Bamaxiang pigs [9], and western pig breeds such as Duroc and Yorkshire [10]. Until now, a total of 6230 QTLs were found on different swine chromosomes for growth and fatness traits in the pig QTL database: (https://www.animalgenome.org/cgi-bin/QTLdb/SS/index, accessed on 21 October 2022). It is noteworthy that a list of important QTLs for body growth and fat deposition traits on Sus scrofa chromosome (SSC)1, 2, 4, 6, and 7 were explored in multiple populations [11,12,13,14], and the candidate genes such as *HMGA1*, *GRM4*, and *PLAG1* were identified at the mapped loci [9,15]. These studies contributed to our understanding of the molecular mechanisms underlying the growth and fatness traits of pigs. 

The objective of the current work was to clarify the genetic architecture of growth and backfat traits in DSE pigs at the finishing stage. To this end, eight phenotypic traits including body weight (BW), body height (BOH), body length (BL), back height (BAH), chest circumference (CC), cannon bone circumference (CBC), abdominal circumference (AC), and backfat thickness (BF) were measured for 265 DSE pigs. Subsequently, using the Geneseek Porcine SNP50 BeadChip array platform, single nucleotide polymorphisms (SNPs) were genotyped in this population. Then, the GWAS analysis was used to detect QTL and to identify candidate genes for those eight traits. Our results offered a foundation for the molecular marker-assisted breeding and improvement of the growth and fatness-related traits in pigs.

## 2. Materials and Methods

### 2.1. Animals and Phenotypic Recording

In current study, all DSE pigs (132 males and 133 females) were derived from the Sipsongpanna breeding farm (a national core DSE pig conservation farm in China). Animals were grouped into different commercial pens and bred under the same nutritional and management conditions. A total of 265 individuals between 390 and 420 days old were selected to measure the growth and fatness-related traits. The recorded phenotypic values were BW, BOH, BAH, CC, BL, CBC, AC, and BF. According to the method applied in previous research [16], six traits (BOH, BAH, CC, BL, CBC, and AC) were measured by a tape or a meter ruler. BAH is the distance between the lowest place of back and ground. The A-mode ultrasonography (Renco lean meter^®^, Minneapolis, MN, USA) was used to determine BF at between 3rd and 4th last ribs (near the dorsal midline at 5 cm) [17]. BW was recorded by a weighing scale. The Ethics Committee of Hunan Agricultural University approved all the experimental procedures in this study (Permit Number: 20210701).

The MEANS procedure of SAS (SAS Institute, Inc., Cary, NC, USA) was used to calculate descriptive statistics for eight traits. Phenotype distribution plots were visualized using R package “ggpubr”. The phenotypic correlations among the eight traits were estimated and visualized by using the “psych” and “corrplot” R packages, respectively [18].

### 2.2. Genotyping and Quality Control

Animal ear tissues were collected for genomic DNA extraction through a typical phenol-chloroform protocol [19]. The concentration and quality of genomic DNA were determined by spectrophotometer Nanodrop 2000 (Thermo Scientific, Waltham, MA, USA) and 1.5% agarose gel electrophoresis. Final concentration of each DNA sample was diluted to 50 ng/μL. Subsequently, 265 DNA samples were genotyped using the Geneseek Porcine 50K SNP Chip (GeneSeek, Lincoln, NE, USA). Plink (version 1.90 beta) [20] was used for quality control of the genotype data with the following parameters: individuals call rate > 90%, SNP call rate > 90%, minor allele frequency (MAF) > 0.05, and Hardy–Weinberg equilibrium (HWE) test *p*-value > 10^−^^5^.

### 2.3. Population Structure and Kinship Identification 

To investigate the population structure of DSE pigs and determine whether principal components (PCs) should be added into GWAS model, we performed a principal component analysis (PCA) based on the filtered SNPs. Briefly, eigenvalues and eigenvectors were calculated by GCTA software [21], and PCA plot was visualized using “ggplot2” R (version 4.2.1) package. In addition, based the genetic relationship matrix (GRM) file, a heat map was visualized to display the level of relatedness for Diannan small ear individuals.

### 2.4. Genome-Wide Association Study (GWAS)

GEMMA (version 0.98.5) [22] was used to perform GWAS analysis between each SNP maker and phenotypic data. The linear mixed model of the GWAS analysis was as follow: y = Zα + Wb + g + e, where y represents the vector of phenotypic values for each individual; α refers to the vector of the fixed effects, in which age (in days), sex, and top two eigenvectors of the principal component were included; Z is the indicator matrix for α; Wb is the marker effect to be tested; g~*N* (0, *A*φ^2^) represents the polygenic effect; A is the kinship matrix achieved prior from the SNPs; and e~*N* (0, *Iσ*^2^) refers to the residual error. Bonferroni correction method was used for the multiple test correction. The threshold values in this study were set based on the Bonferroni correction method. Briefly, *N* (22,146) was the total number of filtered SNPs, the genome-wide significant threshold (2.26 × 10^−^^6^, 0.05/*N*) and genome-wide suggestive level (4.52 × 10^−^^5^, 1/*N*) were used as thresholds. 

### 2.5. GO annotation Analysis of Candidate Genes

To identify the candidate genes of each significant SNP, we searched for annotated genes within 100 Kb upstream or downstream of each significantly associated SNP on pig reference genome (Sscrofa 10.2) (http://asia.ensembl.org/biomart/martview/, accessed on 23 January 2023). In addition, the GO annotation of candidate genes was performed using Gene Ontology Consortium (http://geneontology.org, (accessed on 23 January 2023).

## 3. Results

### 3.1. Genotyping and Phenotypic Statistics

Table 1 summarizes the descriptive statistics of growth and fatness-related traits. The coefficient of variation (CV) ranged from 6.39% (for CBC) to 18.47% (for BF). The distributions of these eight traits were approximately normal distributions(Figure S2A). The Pearson’s correlation analyses between traits showed that seven growth traits (BL, CC, AC, BW, BAH, BOH, and CBC) exhibited positive and significant correlations with each other (0.32 ≤ r ≤ 0.94, *p* < 0.01), whereas BF was weakly correlated with CC (r = 0.19, *p* < 0.001), BW (r = 0.19, *p* < 0.05), and BL (r = 0.18, *p* < 0.001) (Figure S2B).

### 3.2. Identification of SNPs, Principal Component and Kinship Analysis

**Figure 1:**
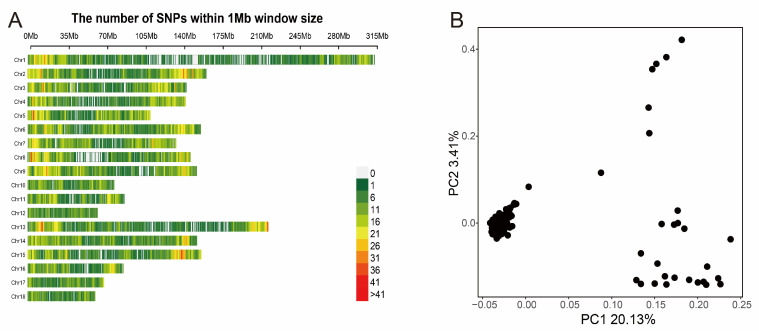
The distribution of SNPs on autosomal chromosomes and the principal component analysis. (**A**) The number of SNPs within a 1 Mb window. (**B**) The PCA-plot of top two principal components.

After genomic mapping and SNP calling, we obtained 22,146 SNPs, in total, in all tested individuals via a series of filters. The density distribution plot of filtered SNPs across Sscrofa genome are shown in Figure 1A. Almost all of the genome’s non-overlapped 1 Mb regions contained SNPs, which indicated that the data was reliable. We also performed the principal component analysis to avoid the false-positive results caused by population stratification, which was considered to be a major threat to the reliability of the GWAS result. The PCA plot (Figure 1B) indicated that the top two principal components should be added to GWAS model as covariates. In addition, a kinship matrix was included in the GWAS model for analysis. The kinship heat map was exhibited in Figure S3.

### 3.3. Genome-Wide Association Results

**Figure 2:**
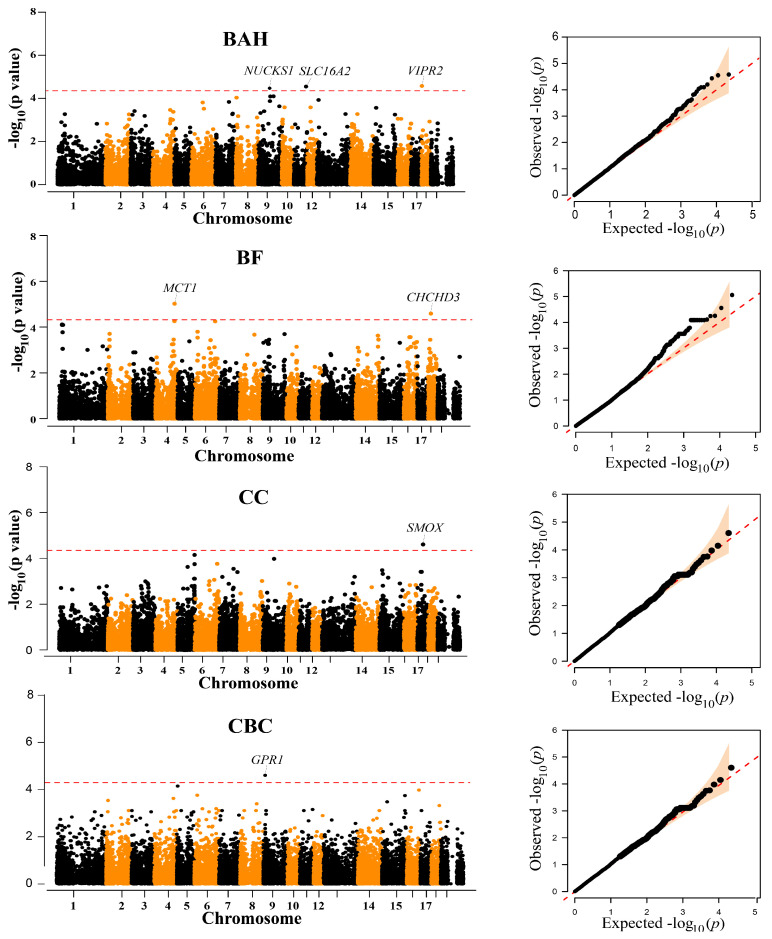
Manhattan and quantile-quantile (QQ) plots of the observed −log10 (*p*-values) for BAH, BF, CC, and CBC in Diannan small-ear (DSE) pigs. The horizontal red dashed lines in the Manhattan plots indicate the suggestive level (4.52 × 10^−5^). The QQ plots show the observed −log_10_-transformed *p*-values (*y*-axis) and the expected −log_10_-transformed *p*-values (*x*-axis).

Traits BAH, BF, CC, and CBC had at least one chromosome-level significant locus (Figure 2, Table 2). Three SNPs, one SNP on SSC9 (WU_10.2_9_72689043), one SNP on SSC11 (ASGA0051858), and one SNP on SSC18 (WU_10.2_18_1597750) were associated with BAH. Additionally, the most significant SNPs associated with BF was ALGA0000014, which explained 2.13% of the phenotypic variation. ALGA0000014 was mapped to the MCT1 gene on chromosome 4 between 117.40 Mb downstream and 117.61 Mb upstream. Another suggestive SNP (WU_10.2_1_778943) for BF was identified at the position 18,121,645 bp on SSC18. Moreover, the INRA0053606 located on SSC17 and MARC0095695 located on SSC15 were associated with CC and CBC, respectively. However, no significant or suggestive SNPs were detected for BW, BOH, BL, and AC (Figure S4).

### 3.4. Comparison with Previously Mapped QTL in Pigs

To verify whether the phenotype-associated SNPs in this study were located in any previously known QTLs, the overlap between our GWAS results and previously reported QTL data were examined using PigQTLdb (Table S1). Compared to the previously reported QTLs, there were no overlapping SNP loci between our study and other reports. In previous studies, a total of 18 SNPs were found to be associated with BF, CC, and CBC of pigs. A total of 14 SNPs on SSC4 or SSC18 were related to BF. As for CC, two QTLs ranging from 18,990,744 bp to 18,997,951 bp were associated with CC on SSC17. Regarding the BAH, no QTLs were found on PigQTLdb.

### 3.5. GO Annotation of Candidate Genes

The results of functional annotation for candidate genes are shown in Table 3. MCT1 was mainly involved in organic hydroxy compound transport (GO:0015850) and lactate transmembrane transporter activity (GO:0015129). The biological process of CHCHD3 and GPR1 was protein insertion into the mitochondrial outer membrane and G protein-coupled receptor internalization, respectively. SLC10A2 and VIPR2 participated in the regulation of bile acid metabolic process (GO:1904251) and hormone secretion (GO:0046879), respectively.

## 4. Discussion

Considerable attention has been given to genomic selection in either western or Chinese indigenous pig breeds, which can improve populations for a set of important traits. In the world, pork accounts for 35% of meat consumption, representing a vital component of human daily diets [23]. The demand for pork has increased due to socioeconomic development in China. It is imperative to elevate pork production and improve its quality, especially for Chinese indigenous pig breeds. DSE pig is a mini-type breed that has excellent meat quality, but has a lower growth rate and small body size. Normally, the DSE breed has been used in disease and pharmaceutics research because it is similar to humans in terms of physiology, anatomy, and pathology [24,25,26]. To our knowledge, there have been limited studies about the genetic improvement of growth and meat quality characters in DSE pigs. It is thus important to increase the research on the relevant economic traits to elevate the economic benefits of this breed. As shown in this study, the Geneseek Porcine 50K SNP chips were used to genotype a sample of the DSE pig population. A genome-wide association analysis was then performed using eight growth and fatness traits and the SNP marker. The findings in this study provide important candidate molecular markers that are associated with growth and fatness traits for DSE pig breeding. Also, this study provides a novel insight into making full use of the local Chinese swine breeds such as the DSE breed in the future.

In this study, seven potential SNPs that surpassed the suggestive level related to four phenotypes (BF, CC, BAH, and CBC). Body measurement trait is closely related to productivity, because it reflects the pig’s growth status. For example, BF is one of the most important fatness traits for pigs. Here, two suggestive SNPs on SSC4 (ALGA0000014) and 18 (WU_10.2_1_778943) were found to be related to BF. The monocarboxylate transporter 1 (*MCT1*, also named *SLC16A1*) gene was closest to ALGA0000014, which plays an important role in the process of glycolysis [27,28]. *MCT1* is an important carrier of short-chain fatty acids and lactate in various tissues [29]. Researchers fed MCT1 ^(+/−)^ mice (C57BL/6J) with a high-fat diet (HFD) and found that MCT1 ^(+/−)^ mice had decreased weight gain, which was largely due to reduced fat accumulation (50.0% less after 9 months of HFD), especially in white adipose and liver tissue [30]. The lactate transport is the key process for adipocyte function. Recently, researchers verified that the mRNA and protein level of *MCT1* were significantly increased during adipocyte differentiation of three different adipocyte cell lines, accompanied by the elevated lactate flux capacity [31]. Coiled-coil-helix-coiled-coil-helix domain containing 3 (*CHCHD3*) was the candidate gene located nearest to the SNP (WU_10.2_1_778943) on SSC18. Suppression of *CHCHD3* could result in tissue undergrowth and cell proliferation defects, suggesting that *CHCHD3* has the capacity of regulating tissue growth [32]. As is known, the three types of adipose tissue related to fatness traits in pigs are intramuscular, subcutaneous, and intermuscular [33]. Subcutaneous fat deposition is associated with meat production, while intramuscular adipose deposition is a key factor for meat quality in commercial pigs. The reports above suggest that *MCT1* and *CHCHD3* are important candidate genes related to fat deposition. Additionally, the results in this study indicated that these two SNPs could be used as key molecular markers for improving the meat quality and/or meat production of pigs.

CC is highly positively related to body weight and is an important indicator of thinness and fatness [34]. The SNP INRA0053606 was found to be significantly associated with CC. The nearest candidate gene was the *SMOX* gene, which is a strong candidate gene significantly related to average daily weight gain (ADG) and ultrasonic measurements of L. dorsi muscle depth (UMD) of Esme sheep [35]. In addition, the enzyme coded by *SMOX* was significantly associated with fiber size, skeletal muscle development, and muscular atrophy and hypertrophy in animals [36,37,38]. These studies indicate that the INRA0053606 in the SMOX gene is an important molecular marker for improving growth traits in pigs.

CBC reflects the physical quality of the livestock, whether it is strong or not [34]. The suggestive SNP (MARC0095695) on SSC15 was in the G protein-coupled receptor 1 (*GPR1*, *CMKLR2*) gene. A previous study showed that *GPR1* was involved in the generation of adipocytes [39]. In addition, the negative regulation of chemerin/GPR1 signaling could inhibit adipogenesis of bone-marrow derived mesenchymal stem cells (BMSCs), which could effectively improve osteoporosis [40]. Interestingly, previous studies demonstrated that the deficiency of *GPR1* contributes to bone loss in mice [41,42]. We speculate that *GPR1* might be related to the powerful leg strength and jumping ability of DSE pigs.

Among the three candidate SNPs related to BAH, WU_10.2_18_1597750 on SSC18 was identified to be close to the vasoactive intestinal peptide receptor 2 (*VIPR2*) gene, also known as *VPAC2R,* which was involved in the insulin secretion process [43]. In a previous study, researchers found that the skeletal muscle mass was increased in predominantly fast-twitch muscle types with the elevation of cAMP levels after injection of VIPR2-selective agonist [44]. In particular, the VIPR2-receptor found in human adipocytes played an important role in controlling fat deposition [45]. Moreover, *VIPR2*-knockout could cause growth inhibition, a decrease in fat mass, and increase in lean mass, as well as a change of IGF-I levels, indicating that *VIPR2* possesses multiple functions in metabolism [46]. Another identified candidate gene for BAH is solute carrier family 10-member 2 (*SLC10A2*, also name as *ASBT*). As reported, *SLC10A2* is associated with body weight gain and could improve insulin sensitivity [47]. It was reported that blocking *SLC10A2* decreased serum triglyceride (TG) and hepatic production of TG, hinting at the vital role in regulation of lipid metabolism [48]. The nuclear casein kinase and cyclin dependent kinase substrate 1 (*NUCKS1*) gene was identified near the SNP WU_10.2_9_72689043. A recent GWAS study indicated that *NUCKS1* is associated with increases in height [49]. When examining the PigQTLdb, we noted that no QTLs related to BAH were included in the database. This may be because researchers usually study BOH instead of BAH. Our findings suggest that BAH is another important growth trait that could be taken into account during the molecular breeding of pigs.

In the current study, only seven candidate SNP loci were identified with suggestive thresholds by the genome-wide association study, indicating the limitations of this study such as limited sample size and genotyping platform. Compared with other genotyping methods, such as whole genome resequencing analysis, the SNP chip data can only offer limited information throughout the whole genome. The SNP markers of the Geneseek Porcine 50K SNP Chip were mainly designed based on the SNP markers identified from western pig breeds. Therefore, some important information in the Chinese native pig breed such as the DSE pig may be ignored in this platform because of the different genetic background among different breeds. Moreover, the statistical power for candidate gene discovery could have been limited by small sample size. Therefore, it is necessary to verify these candidate loci’s genetic effects in a larger samples and other pig breeds. Additionally, further investigation on the functional mechanism of these important candidate genes identified in this research is necessary. Other strategies, such as whole-genome sequencing and other SNP chips designed specifically for Chinese native breeds of pigs, will be meaningful to increase the statistic power of the GWAS and elucidate the underlying function of specific genes.

## 5. Conclusions

To summarize, a total of seven SNPs were found to be significantly associated with BAH, BF, CC, and CBC in DSE pigs by GWAS at the suggestive significance level. Based on gene annotation analysis, these phenotype-associated SNPs were mapped to the important candidate genes, such as *MCT1*, *SMOX*, and *VIPR2*, which are mainly involved with adipocyte differentiation, skeletal muscle development, and average daily weight gain. These results can contribute to understanding the genetic basis of pig growth and development and provide potential candidate molecular markers for DSE pig-breeding programs.

## Figures and Tables

**Table 1:** Descriptive statistics of phenotypic data in Diannan small-ear (DSE) pigs for growth and fatness traits.

Traits	*N*	Mean ± SE ^1^	Max	Min	CV (%) ^2^
BW (kg)	265	42.12 ± 0.60	63	26.5	17.11
BOH (cm)	265	49.00 ± 0.29	57.5	41.1	7.23
BAH (cm)	265	47.50 ± 0.29	55.2	39.5	7.49
CC (cm)	265	85.65 ± 0.57	100.2	70.5	7.99
BL (cm)	265	89.42 ± 0.54	104.3	73.2	7.22
CBC (cm)	265	12.60 ± 0.06	15	10.2	6.39
AC (cm)	265	96.30 ± 0.63	100.45	76.2	7.93
BF (cm)	265	23.00 ± 0.35	37.15	14.5	18.47

Abbreviations: BW, body weight; BOH, body height; BAH, back height; CC, chest circumference; BL, body length; CBC, cannon bone circumference; AC, abdominal circumference; BF, backfat thickness. ^1^ Standard error. ^2^ Coefficient of variation.

**Table 2:** Single nucleotide polymorphism markers with suggestive thresholds identified by the genome-wide association study.

Trait Name	SSC ^1^	SNP ID	Position (bp)	*p*-Value	MAF ^2^	*β* ^3^	Distance ^4^ (bp)	PVE ^5^	Candidate Gene
BAH	9	WU_10.2_9_72689043	72,689,043	3.68 × 10^−5^	0.41 (A/G)	−1.6	20,333	1.85%	*NUCKS1*
BAH	11	ASGA0051858	79,182,748	2.83 × 10^−5^	0.30 (G/A)	2.07	565,366	1.85%	*SLC10A2*
BAH	18	WU_10.2_18_1597750	1,597,750	2.65 × 10^−5^	0.13 (A/G)	2.55	934,398	1.87%	*VIPR2*
BF	4	ALGA0000014	117,457,281	8.72 × 10^−6^	0.03 (G/A)	6.37	146,353	2.13%	*MCT1*
BF	18	WU_10.2_1_778943	18,121,645	2.75 × 10^−5^	0.10 (A/G)	4.17	713,183	1.87%	*CHCHD3*
CC	17	INRA0053606	35,923,016	2.48 × 10^−5^	0.09 (A/G)	6.19	36,085	1.89%	*SMOX*
CBC	15	MARC0095695	120,257,203	2.02 × 10^−5^	(0.25 C/A)	−0.53	756,834	2.03%	*GPR1*

BAH, back height; BF, backfat thickness; CC, chest circumference; CBC, cannon bone circumference. ^1^ Sus scrofa chromosome. ^2^ allele frequency of first listed marker. ^3^ allele substitution effect. ^4^ the distance between the SNP and the nearest candidate gene. ^5^ phenotypic variation explained.

**Table 3:** The description and GO annotation of the genes within 100 kb upstream and downstream of the significant SNP.

Genes	Descriptions	GO Annotation
*MCT1*	monocarboxylate transporter 1	BP: Organic hydroxy compound transport (GO:0015850); MF: Lactate transmembrane transporter activity (GO:0015129);
*CHCHD3*	Coiled-coil-helix-coiled-coil-helix domain-containing protein 3 membrane	BP: Protein insertion into mitochondrial outer membrane (GO:0045040); MF: NADH dehydrogenase (ubiquinone) activity (GO:0008137); CC: SAM complex (GO:0001401);
*GPR1*	G-protein coupled receptor 1	BP: G protein-coupled receptor internalization (GO:0002031); MF: G protein-coupled receptor binding (GO: 0001664);
*SLC10A2*	Solute carrier family 10 (sodium/bile acid cotransporter), member 2;	BP: Regulation of bile acid metabolic process (GO:1904251); MF: Bile acid transmembrane transporter activity (GO:0015125);
*VIPR2*	Vasoactive intestinal polypeptide receptor 2	BP: Hormone secretion (GO:0046879); MF: Glucagon receptor binding (GO:0031769); CC: Extracellular space (GO:0005615);
*SMOX*	Spermine oxidase	BP: Polyamine catabolic process (GO:0006598); CC: Spermidine binding (GO:0019809);
*NUCKS1*	Nuclear casein kinase and cyclin-dependent kinase substrate 1	BP: interstrand cross-link repair (GO:0036297); CC: cytoplasm (GO:0005737); MF: single-stranded DNA binding (GO:0003697);

## Data Availability

The data presented in this study are available on request from the corresponding author.

## Supplementary Materials

The following supporting information can be downloaded at: https://www.mdpi.com/article/10.3390/ani13091571/s1, Figure S1: Diannan small-ear (DSE) pig. Table S1: Data of significant SNPs with previously reported QTLs from the pig QTL database for growth and fatness-related traits. Figure S2: A. The normal distribution plot of eight growth and fatness traits for DSE pigs. B. Correlation analyses between eight traits for DSE pigs. The correlation coefficients at the bottom left are calculated by Pearson’s correlation analysis. The significant correlations are presented as asterisks (*, *p* < 0.05; **, *p* < 0.01). Abbreviations: BW, body weight; BOH, body height; BAH, back height; CC, chest circumference; BL, body length; CBC, cannon bone circumference; AC, abdominal circumference; BF, backfat thickness. The size and color of red balls represent the magnitude of the correlation coefficients. Figure S3: The kinship relationships of DSE pigs. The average value for genomic relationship matrix was 0.98, while the maximum and minimum value were 0.185 and −0.179, respectively; Figure S4. Manhattan and quantile-quantile (QQ) plots of the observed −log_10_ (*p*-values) for BW, BOH, BL, and AC in Diannan small-ear (DSE) pigs. The horizontal red dashed lines in the Manhattan plots indicate the suggestive level (4.52 × 10^−5^). The QQ plots show the observed −log_10_-transformed *p*-values (*y*-axis) and the expected −log_10_-transformed *p*-values (*x*-axis).
