# Supplementary material for: Genome-Wide Association Analysis Identifies Genomic Regions and Candidate Genes for Growth and Fatness Traits in Diannan Small-Ear (DSE) Pigs
Source: Animals (Basel). 2023 May 8;13(9):1571. doi: 10.3390/ani13091571 (PMC10177038; doi:10.3390/ani13091571)
Supplement: Supplementary file 1 [file animals-13-01571-s001.zip › Figures S1¿CS4.pdf]

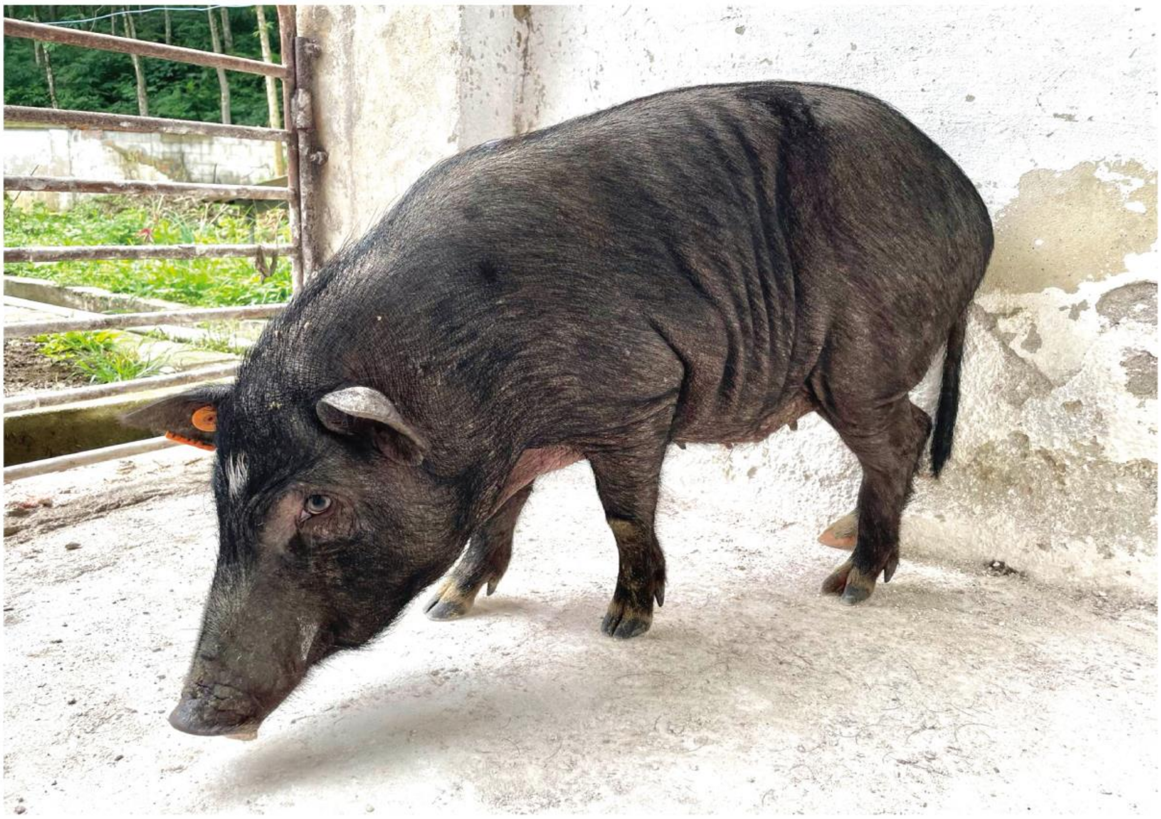

Figure S1. Diannan small-ear (DSE) pig.

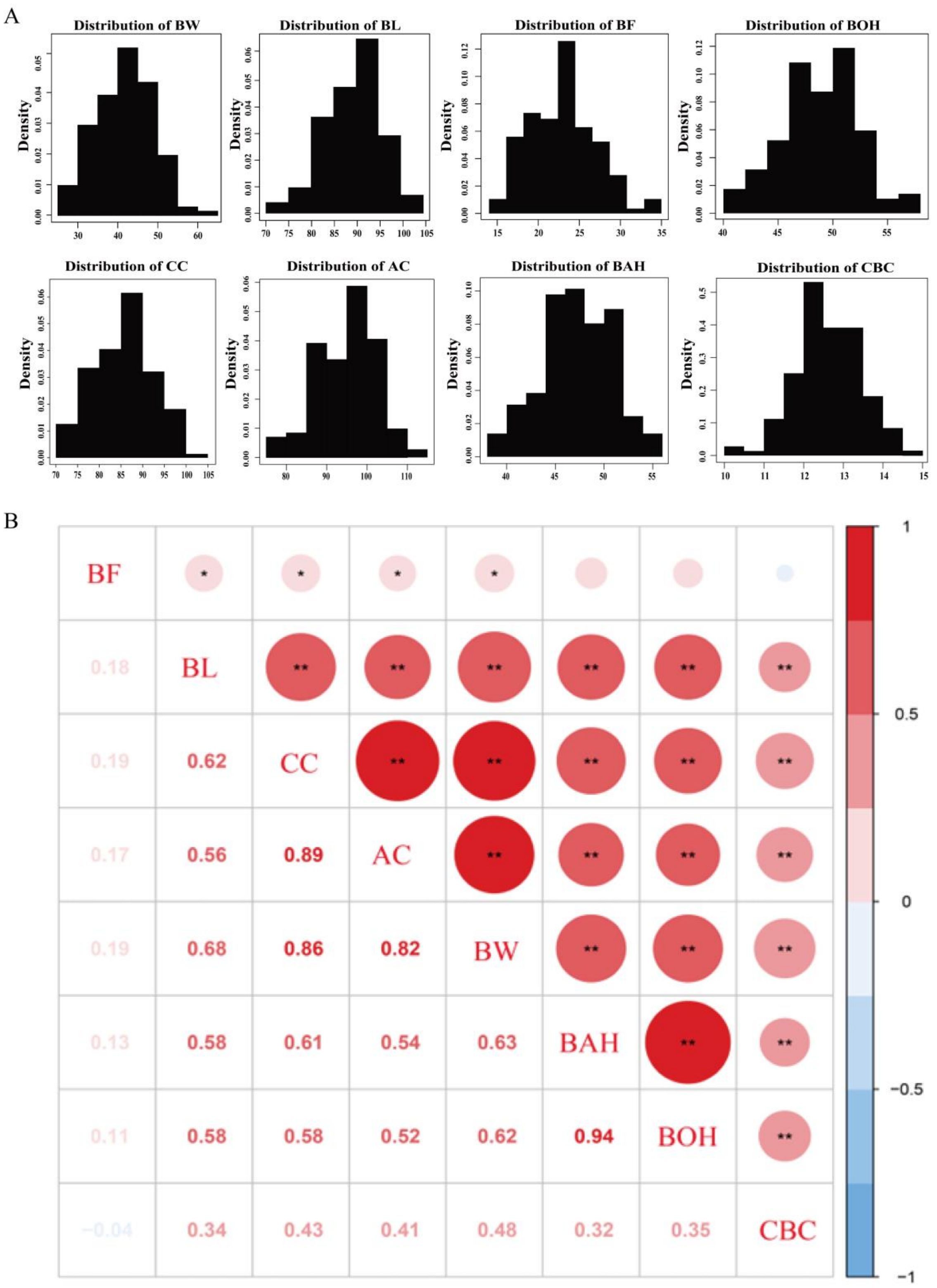

Figure S2. (**A**) The normal distribution plot of eight growth and fatness traits for DSE pigs. (**B**) Correlation analyses between eight traits for DSE pigs. The correlation coefficients at the bottom left are calculated by Pearson's correlation analysis. The significant correlations are presented as asterisks (*, $p < 0.05$; **, $p < 0.01$). Abbreviations: BW, body weight; BOH, body height; BAH, back height; CC, chest circumference; BL, body length; CBC, cannon bone circumference; AC, abdominal circumference; BF, backfat thickness. The size and color of red balls represent the magnitude of the correlation coefficients.

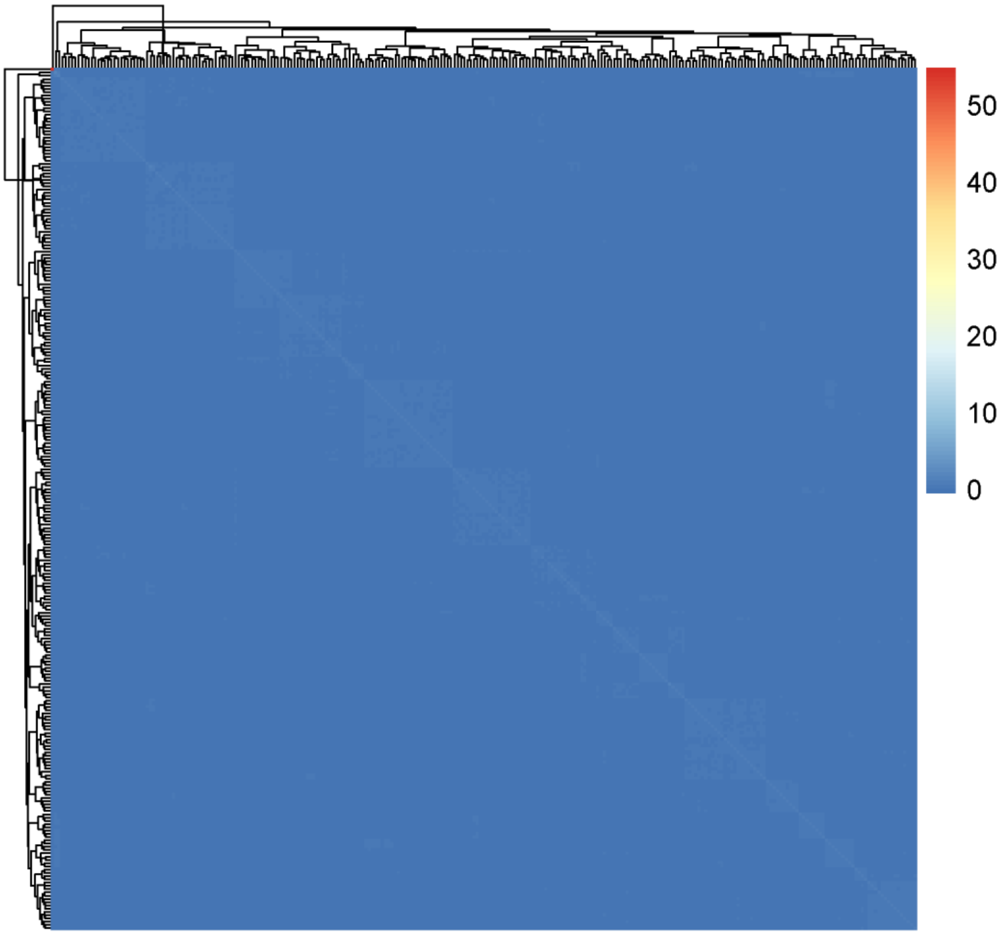

Figure S3. The kinship relationships of DSE pigs. The average value for genomic relationship matrix was 0.98, while the maximum and minimum value were 0.185 and −0.179, respectively.

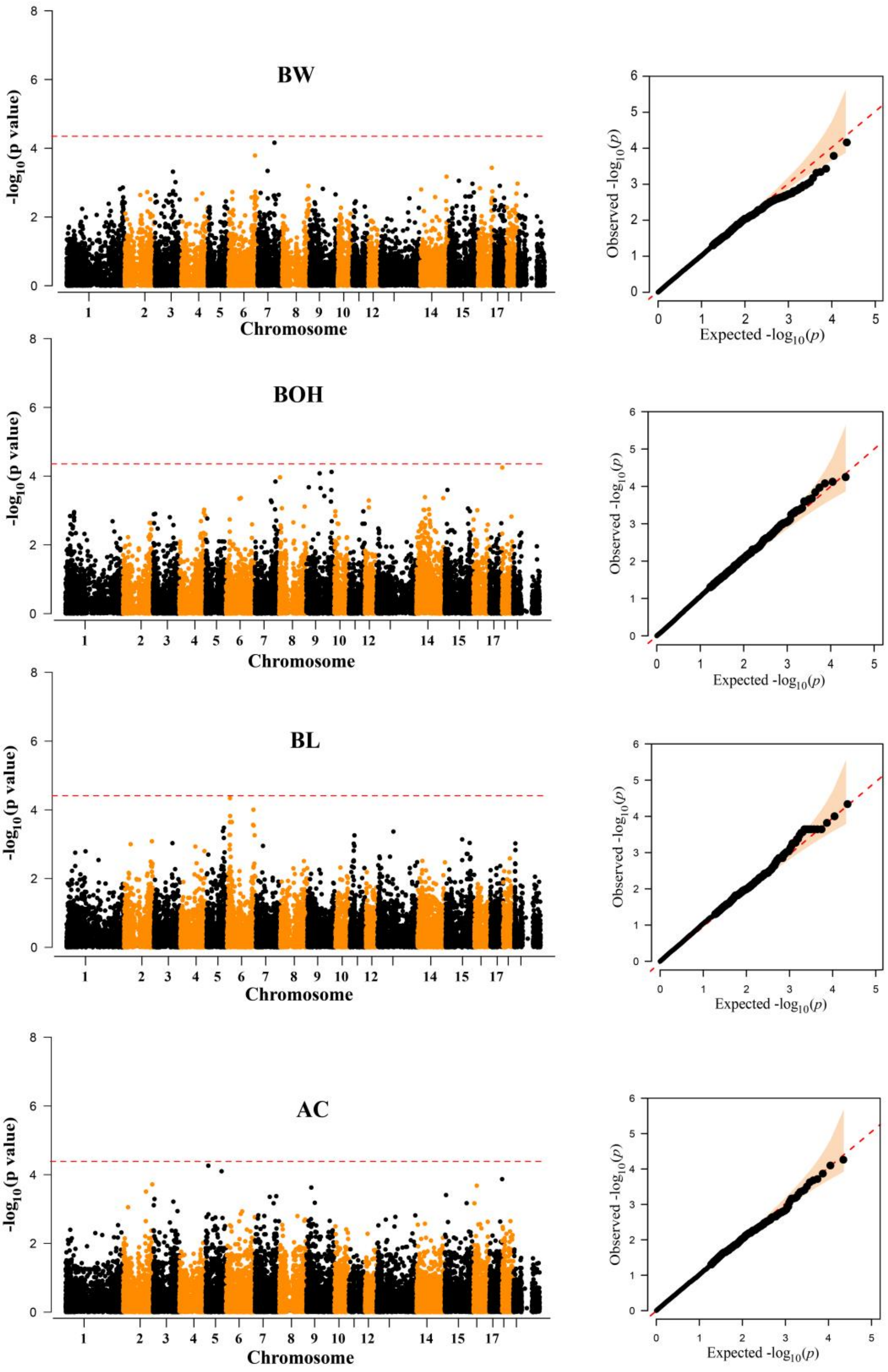

Figure S4. Manhattan and quantile-quantile (QQ) plots of the observed $-\log_{10}$ (p-values) for BW, BOH, BL, and AC in Diannan small-ear (DSE) pigs. The horizontal red dashed lines in the Manhattan plots indicate the suggestive level (4.52×10^{-5}). The QQ plots show the observed $-\log_{10}$-transformed p-values (y-axis) and the expected $-\log_{10}$-transformed p-values (x-axis).
